# Characteristics of the Health‐Associated Oral Microbiome in Young Nonhuman Primates

**DOI:** 10.1111/omi.70033

**Published:** 2026-05-16

**Authors:** J. L. Ebersole, O. A. Gonzalez

**Affiliations:** ^1^ Department of Biomedical Sciences, School of Dental Medicine University of Nevada Las Vegas, Las Vegas, Nevada; ^2^ Department of Oral Health Sciences and Center for Oral Health Research, College of Dentistry University of Kentucky, Lexington, Kentucky

**Keywords:** aging, nonhuman primates, oral microbiome, periodontal health

## Abstract

Nonhuman primates have been shown to develop periodontitis with clinical and demographic features similar to humans. This preclinical disease model is well‐positioned to advance the elucidation of novel biologic‐based preventive and therapeutic approaches to controlling periodontitis, a chronic infection and immunoinflammatory disease. In this analysis, the oral microbiomes of younger (10–19 human years; *n* = 30) nonhuman primates are compared for matriline (an indicator of heritability), sex, and age variations. A holistic assessment of the microbiome similarities/differences suggested two primary conclusions in this younger group of orally healthy nonhuman primates. First, at the microbiome level of phyla, orders, and families, the ecology was relatively similar across sexes, matrilines, and age groups. However, at the genus level, matriline, sex, or age differences were observed. Of interest was that the principal differential genus proportions with matriline and age were similar, but somewhat unique with the sex comparison. These genus differences encompassed microorganisms generally considered as human commensals, albeit *Fusobacterium, Tannerella*, and *Treponema* genera did show some variation. The second, broader observation was the rather extensive species variation across these nonhuman primates. Nevertheless, the data could define a “core microbial species” pattern that included species across the *Actinobiota, Bacteroidota, Desulfobacterota, Firmicutes/Bacillota, Fusobacteriota, Proteobacteriota*, and *Spirochaetota* phyla. The results provide seminal details of the oral microbiome in this disease model and underpin the ability to elucidate specific microbial changes that can occur related to early‐life oral environmental stimuli that may presage a greater risk for periodontitis in adulthood.

## Graphical abstract

The oral microbiome of healthy younger nonhuman primates, as a preclinical model of the human oral cavity, was evaluated. The results provide seminal details of the oral microbiome in this disease model to elucidate specific microbial changes. These differences occur in early‐life oral environmental stimuli and may presage a greater risk for periodontitis in adulthood.

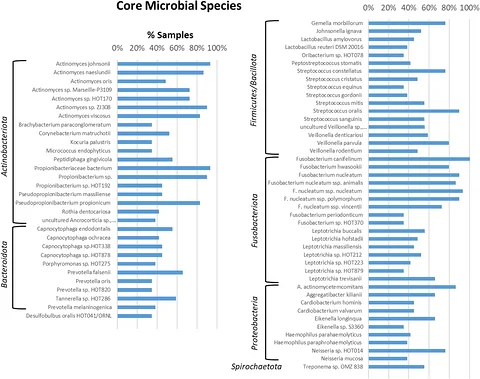

## 1 Introduction

Periodontal disease is a global noncommunicable disease that primarily affects adults after the age of 35 years (Collaborators 2025; Eke et al. 2016). However, a more limited prevalence of periodontitis is identified in younger individuals, generally representing a more aggressive form of the disease, with differences in both host and microbial aspects of the disease process (Catunda et al. 2019). The adult disease has been linked to a dysbiotic microbiome and dysregulated host immunoinflammatory response (Ebersole et al. 2017; Joseph and Curtis 2021; Nibali et al. 2022; Sedghi et al. 2021; Yama et al. 2023). Observations document a progressive alteration of the microbial components comprising disease‐associated biofilms, as well as modified local host responses to these biofilms, which contribute to the tissue‐destructive biology of periodontal lesions. Additionally, results from various types of genetic studies have implicated a level of genetic impact on individual host susceptibility related to the extent and severity of the disease (Agler and Divaris 2020; Nibali et al. 2019; Vaithilingam et al. 2014; Zhang et al. 2020).

These risks include the identification of enhanced susceptibility for disease within families (Diehl et al. 2003; Hodge et al. 2000; Michalowicz 1994; Nibali et al. 2020; Schenkein and Van Dyke 1994; Sima et al. 2015). This observation has been explained by the potential transmission of more pathogenic biofilm microbes (Asikainen et al. 1986; Monteiro et al. 2021) and the heritability of specific genetic and/or behavioral risk profiles (Caetano et al. 2022; Hart 1994; Kinane et al. 1999; Michalowicz 1994).

Examination of the bacteria generally described as periodontal pathogens has ascertained their presence even in the oral microbiome of young individuals in the absence of clinical measures of disease (Bimstein and Ebersole 1989; Bimstein et al. 2004; Monteiro et al. 2021; Okada et al. 2004). Additionally, prevailing data define the existence of “risk genes,” even in young individuals, that occur decades before exhibiting periodontitis. Moreover, while clinical reports clearly define that a large proportion of children and adolescents express robust gingival inflammation and bleeding (Bimstein et al. 2013; Dibart 1997; Kinane and Hodge 2001; Modeer and Wondimu 2000), as well as harboring periodontal pathogens (Gafan et al. 2004; Kisby et al. 1989; Okada et al. 2004), they generally do not demonstrate a high frequency of irreversible tissue‐destructive disease. Thus, the inference is that this inflammatory response differs from the process resulting in the clinical inflammation in adults, and may provide information regarding biologic features of less or non‐destructive inflammation.

The nonhuman primate has been shown to express periodontitis that is clinically, microbiologically, and immunologically similar to the human disease, and the disease is increased with age, obesity, and in males, similar to humans (Ebersole et al. 2025). We have also reported on the identification of matrilines from within the colony of *Macaca mulatta* derived from the Cayo Santiago, Puerto Rico primate facility that has existed for over 85 years. The matrilines vary in presenting members over 8–9 generations, who appear more resistant or susceptible to periodontitis (Ebersole et al. 2023; Ebersole et al. 2023a; Ebersole et al. 2019; Gonzalez et al. 2016). As such, we conducted a cross‐sectional study of young nonhuman primates derived from the matrilines to evaluate the autochthonous oral microbiome associated with periodontal health. The objective was to assess natural variations in the microbiome that potentially reflect matriline differences, compared with those of age and sex disparities, and the potential for increased susceptibility to periodontal disease in adult nonhuman primates.

## 2 Materials and Methods

### 2.1 Nonhuman Primate Model and Oral Clinical Evaluation

Rhesus monkeys (*Macaca mulatta*) housed at the Caribbean Primate Research Center (CPRC) at Sabana Seca, Puerto Rico, were used in these studies. Thirty healthy nonhuman primates were enlisted with 14 females and 16 males from 3.1–5.5 years of age [young ∼10–12 human years (*n* = 16); young adult (YAdu) ∼13–19 (*n* = 14)]; 11 from Periodontitis Resistant (PDR) matrilines and 19 from Periodontitis Susceptible (PDS) matrilines, as we have previously reported (Ebersole et al. 2023; Ebersole et al. 2023a; Ebersole et al. 2019). A protocol approved by the Institutional Animal Care and Use Committee (IACUC) of the University of Puerto Rico enabled anesthetized nonhuman primates to be examined for clinical measures of periodontal parameters, including probing pocket depth (PPD), and bleeding on probing (BOP), as we have described previously (Ebersole et al. 2008a,b). A full mouth examination, excluding 3^rd^ molars and canines, using a Maryland probe on the facial aspect of the teeth, for example, 2 interproximal sites per tooth (mesio‐ and disto‐buccal), identified the 30 nonhuman primates as periodontally healthy, classified by a mean BOP of ≤1.0 and mean PPD of ≤3.0. The methods were carried out following all relevant regulations for the use of nonhuman primates, based on ARRIVE guidelines (https://arriveguidelines.org/resources/author‐checklists).

The nonhuman primates are typically fed a 20% protein, 5% fat, and 10% fiber commercial monkey diet (diet 8773, Teklad NIB primate diet modified: Harlan Teklad). The diet is supplemented with fruits and vegetables, and water is provided ad libitum in an enclosed corral setting.

### 2.2 Microbiome and Data Analysis

Bacterial plaque samples were collected using curettes from three healthy subgingival sites for molecular microbiome analysis conducted by Zymo Research (Irvine, CA). The pooled samples were derived from a first molar tooth in 3 different quadrants from each monkey. The plaque was handled and managed for sequencing (Kozich et al. 2013) to determine the total composition of the oral microbiota from each sample, as we have described previously (Ebersole et al. 2020; Kirakodu et al. 2019). The raw data is being deposited. Statistical differences of bacterial OTU (Operational Taxonomic Units) were determined with a Mann–Whitney U test at least at *p* < 0.05.

The samples were processed and analyzed with the ZymoBIOMICS Shotgun Metagenomic Sequencing Service for Microbiome Analysis (Zymo Research, Irvine, CA). The ZymoBIOMICS‐96 MagBead DNA Kit (D4302, Zymo Research, Irvine, CA) was used to extract DNA using an automated platform. Genomic DNA samples were profiled with shotgun metagenomic sequencing. Sequencing libraries were prepared with the Illumina DNA Prep Kit (Illumina, San Diego, CA) following the manufacturer's protocol using 10 bp unique dual indexes. All libraries were quantified with Qubit (Thermo Fisher Scientific) and then pooled together by equal abundance. The final pool was quantified using qPCR. The final library was sequenced on either the Illumina NextSeq 2000 or the Illumina NovaSeq X.

Bioinformatics analysis provided by Zymo included raw sequence reads that were trimmed to remove low‐quality fractions and adapters with Trimmomatic‐0.33 (Bolger et al. 2014): quality trimming by sliding window with 6 bp window size and a quality cutoff of 20, and reads with a size smaller than 70 bp were removed. After that, host‐derived reads were removed using Kraken2 (Wood et al. 2019) against some common eukaryotic host genomes. Low‐diversity reads were detected and removed using sdust (https://github.com/lh3/sdust). The surviving reads were subjected to further taxonomy and functional analyses as follows. Microbial composition was profiled using Sourmash (Pierce et al. 2019). The GTDB species representative database (RS207) was used for bacterial and archaeal identification. Reads were mapped back to the genomes identified by Sourmash using BWA‐MEM (Li et al. 2013), and the microbial abundance was determined based on the counts of mapped reads. The resulting taxonomy and abundance information were further analyzed: (1) to perform alpha‐ and beta‐diversity analyses; (2) to create microbial composition barplots with QIIME (Kuczynski et al. 2012); and (3) to create taxa abundance heatmaps with hierarchical clustering (based on Bray–Curtis dissimilarity).

## 3 Results

### 3.1 Clinical Features of Oral Health in Young Nonhuman Primates

Figure 1A summarizes the clinical features of the 30 nonhuman primates stratified into periodontitis‐resistant (PDR) and periodontitis‐susceptible (PDS) matrilines. As noted, the mouth mean PPD and mean BOP index were low in all nonhuman primates, with no differences between the PDR and PDS groups. Evaluating these measures for the frequency of sites with a PPD >2 mm or BOP>0 identified some variation across the nonhuman primates, although with little difference between PDR and PDS matrilineal derivation. Figure 1B stratifies these nonhuman primates based on sex. In this assessment, it appeared that females demonstrated a somewhat greater extent of BOP than males, with no differences in PPD. Grouping the nonhuman primates by age (Figure 1C) shows similar clinical features, although the frequency of sites with PPD >2 mm or demonstrating BOP was somewhat higher in the YAdu nonhuman primates.

FIGURE 1Clinical features of bleeding on probing (BOP) and probing pocket depth (PPD) in the nonhuman primates stratified by (A) matriline (e.g., heritability), (B) sex, and (C) age.

**Figure:**
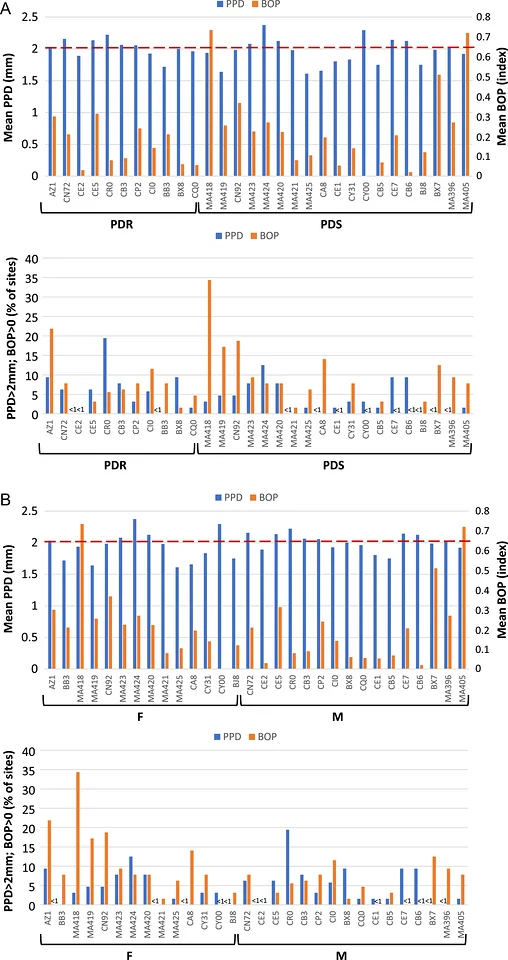

**Figure:**
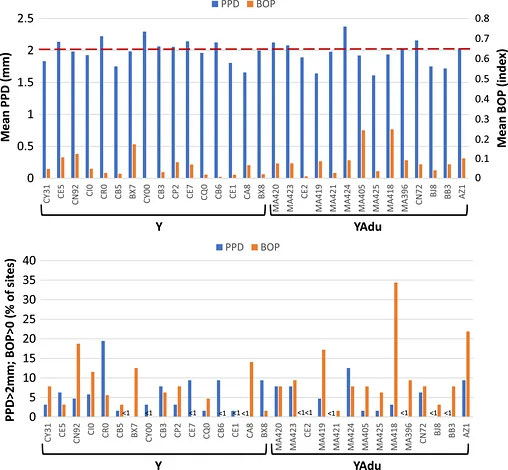

### 3.2 Oral Microbiome Features of Healthy Young Nonhuman Primates

Figure S1 provides an overview of the microbiome features across samples. The Shannon alpha diversity index measures differences in species richness across samples, showing little variation with respect to the animals' matrilineal derivation. However, females and young adult (YAdu) animals exhibited elevated alpha diversity, reflecting increased microbial diversity in their oral microbiomes. The Bray–Curtis assessment of beta diversity showed clear differences in the individual nonhuman primate microbiomes, with approximately 45% of species variation explained by individual sample differences. Nevertheless, no clear patterns were identified based on matriline, sex, or age as stratifiers.

**FIGURE 2:**
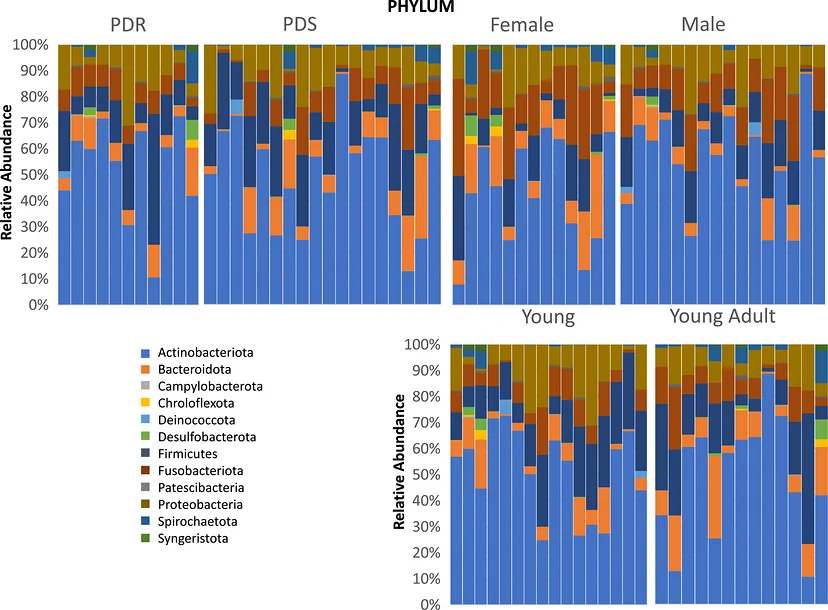
Relative abundance of microbial phyla in the oral microbiome stratified by matriline (PDR/PDS), sex (female/male), and age (young, young adult).

Figure 2 demonstrates the phylum distribution between the PDR and PDS, or based on the sex or age of the nonhuman primates. Increases in *Bacteroidota* and *Spirochaetota* were noted in the PDS samples. Small differences were noted in the relative abundance of individual phyla, with the *Actinobacteriota* increased in males and *Bacteroidota* and *Spirochaetota* elevated in females. Generally, the phyla distribution based on age in the Y versus the YAdu nonhuman primates showed a higher abundance of *Actinobacteriota* and *Proteobacteriota*, with lower levels of *Bacterioidota* and *Spirochaetota* in the Y group.

The relative abundance of the microbial orders is presented in Figure S2, with only the *Veillonellales* significantly increased in PDS. *Propionibacterales, Anaerolineales, Cardobaceriales, Desulfovibrionales, Synergistales*, and *TANB77* were all increased somewhat in the PDR samples. Sex‐based differences showed elevated *Actinomycetales* and *Burholderiales* in males, with *Bacteroidales, Lacnospirales, Peptostreptococcales, Treponematales, Oscillospirales*, and *Tisserellales* all significantly increased in the female samples. *Veillonellales, Desulfovibrionales, Erysipellatrichales, Saccharimonadales, Selenomonadales, Syntergistales*, and *TANB77* were also elevated in the female versus male samples. The effect of age on the microbiome is also demonstrated in Figure S2. Significantly elevated abundance of *Burholderiales* was noted in the Y group, while *Treponemales* and *Tisserellales* were increased in the YAdu samples. The results suggested that, in this younger, orally healthy group, sex influenced the microbiome more than matrilineal or age‐related factors.

Stratifying the microbiome at the family level demonstrated more distinctive differences between the PDR and PDS samples, as shown in Figure S3. Distinguishing differences were noted for *Bacteroidaceae, Leptotrichiaceae, Propionibacteriaceae*, and *Veillonellaceae* between the matrilineal monkey microbiomes. In contrast, male nonhuman primates showed elevations in the relative abundance of *Leptotrichiaceae, Micrococcaceae, Mycobacteriaceae*, and *Neisseriaceae*. The female samples demonstrated increased relative abundance of *Bacteroidaceae, Fusobacteriaceae*, and *Veillonellaceae*. Based on the age grouping of Y and YAdu nonhuman primates, differences in the microbial family distribution (Figure S3) showed elevations in *Bacteroideaceae* and *Fusobacteriaceae*, and decreased levels of *Neisseriaceae* and *Veillonellaceae* in the YAdu samples. Thus, more specific taxonomic classifications resulted in notable differences among sex, matriline, and age‐derived samples.

**FIGURE 3:**
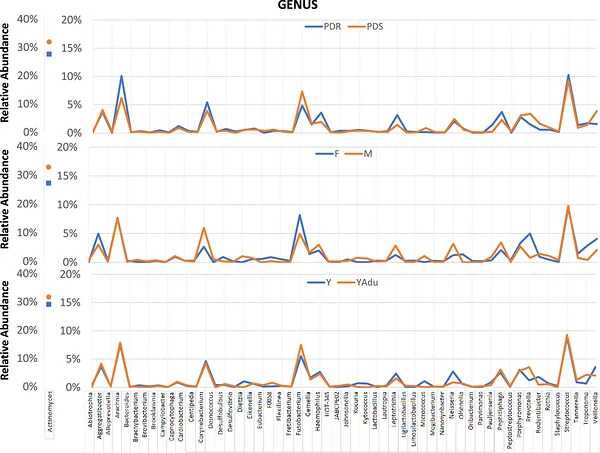
Relative abundance of microbial genera in the oral microbiome stratified by matriline (PDR/PDS), sex (female/male), and age (young, young adult). The values denote the mean relative abundance across all 30 samples.

Genus‐level discrimination of the microbiomes exposed an even greater frequency of differences related to heritability or sex (Figure 3). The PDR samples demonstrated increased abundance of *Arachnia, Corynebacterium, Haemophilus, Leptotrichia*, *Pauljensenia*, and *Peptidophaga*, while the PDS samples exhibited elevations in *Fusobacterium, Prevotella*, and *Veillonella*. Stratification of the genus distribution, based on sex, showed elevations of *Corynebacterium, Leptotrichia, Neisseria*, and *Peptidophaga*, in male samples, with *Aggregatibacter, Fusobacterium, Olsenella, Prevotella, Treponema*, and *Veillonella* increased in the samples from female nonhuman primates. Evaluation of genus‐level differences showed a pattern in the Y and YAdu that more paralleled the ecology, comparing the PDR and PDS animal samples. The YAdu microbiome showed increased abundance of *Fusobacterium, Prevotella*, and *Treponema*, while the Y samples showed increases in *Neisseria, Rodentibacter*, and *Veillonella*. These differences suggest that even in younger nonhuman primates, increasing age is associated with an increase in potential pathogens in the oral cavity, similar to observations in human disease.

**FIGURE 4:**
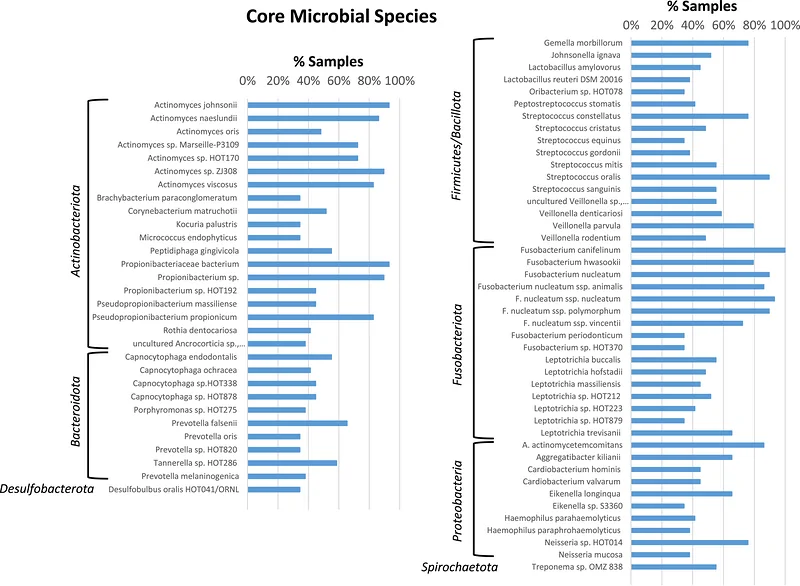
Identification of core microbial species across the microbiomes from the 30 nonhuman primates. Those species included were detected in at least ⅓ of the animal samples. Species are identified and organized by phyla.

Examination of the species distribution across all samples identified a core set of microbial species detected in one‐third or more of the samples. Figure 4 displays an array of 74 species comprising this core oral microbiome from orally healthy young nonhuman primates. The striking feature of this tabulation is that the microbiome in healthy young nonhuman primates contains species associated with periodontitis, including *Porphyromonas, Tannerella, Fusobacterium*, and *Treponema*, suggesting that appropriate local and/or systemic environmental conditions could drive the oral ecology to dysbiosis. This alteration in the microbiome through ligature‐induced experimental periodontitis has been shown in studies of young primates (Ebersole et al. 2020; Kirakodu et al. 2019).

### 3.3 Microbial Species Distribution in Young Nonhuman Primates

**FIGURE 5:**
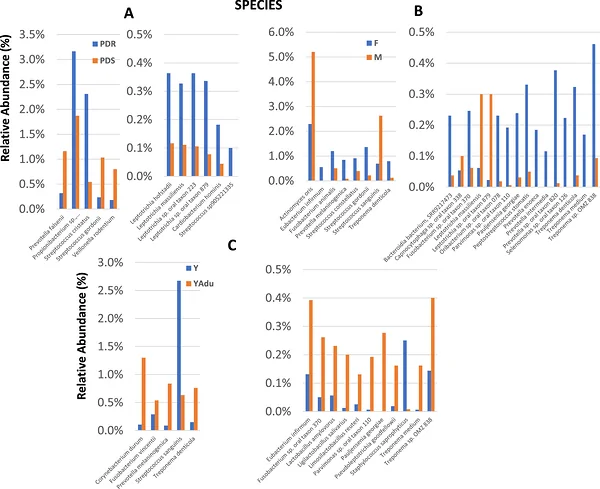
Relative abundance of microbial species in the oral microbiome stratified by (A) matriline (PDR/PDS), (B) sex (female/male), and (C) age (young, young adult). Only those species that were significantly different at *p* < 0.05 are presented.

Figure 5A summarizes the relative abundance of dominant oral microbiome species with significant differences comparing the matrilineal derivation of the nonhuman primates. Examination of the higher‐abundance species showed both *Propionibacterium* sp. ADM1333394 and *S. cristatus* were significantly elevated in PDR and *Prev. falsenii*, *S. gordonii*, and *V. rodentium* were elevated in PDS samples. OTUs with lower abundance also showed variations between the samples, albeit only *L. hofstadii, L. massillesis, Leptotrichia* sp. oral taxon 223, *Leptotrichia* sp. oral taxon 879, *Cardiobacterium hominis*, and *Streptococcus* sp905221335 were significantly increased in PDR samples.

Sex stratification of species distribution in the samples is shown in Figure 5B. In this comparison, many more significant differences were noted. Female nonhuman primates demonstrated a high relative abundance of *Fusobacterium animalis, Eubacterium infirmum, Prev. melaninogenica, S. gordonii*, and *T. denticola*, while *A. oris* and *S. sanguinis* were increased in male samples. In lower abundance species, 13 species were significantly elevated in females, with only *L. massillenesis, Capnocytophaga* sp. oral taxon 338, and *Leptotrichia* sp. oral taxon 879 increased in males.

Figure 5C displays age effects on the microbial species distribution of nonhuman primates. Significant variations in diverse species were observed between the age groups, as observed with both matriline and sex. The majority of these differences were found with significantly elevated levels in the YAdu nonhuman primates. Only *Propionibacteriaceae* bacterium SRR9217400, *S. sanguinis, V. parvula, and Pseudoleptotrichia saprophyticus* were significantly increased in the Y samples.

Figure 6 summarizes the oral microbiome members that showed at least a twofold relative abundance difference based on matriline, sex, or age. Figure 6A compares PDR and PDS samples, with the OTUs elevated in the PDR samples appearing skewed toward many Gram‐positive bacteria and Gram‐negative commensals, such as *Leptotrichia* species. Interestingly, elevations in some *Treponema* and *Tannerella* species, as well as sulfate‐reducing species (e.g., *Desulfobulbus, Desulfovibrio*), did support components of the microbiome that could contribute to pathogenic changes under appropriate circumstances. Variations in the PDS microbial distribution are also shown in Figure 6A. In this case, elevations in numerous *Porphyromonas, Prevotella*, and *Fusobacterium* species were observed. Also of interest was the array of *Rodentibacter* species that showed elevated levels and have been identified as important infection agents in rodents.

FIGURE 6Relative abundance of microbial species in the oral microbiome stratified by (A) matriline (PDR/PDS), (B) sex (female/male), and (C) age (young, young adult). Those species included showed at least a twofold difference between the different sample groups that were compared. The red dashed line separates those elevated in PDR, F (female), or Y (young) versus those elevated in PDS, M (male) or YAdu (young adult).

**Figure:**
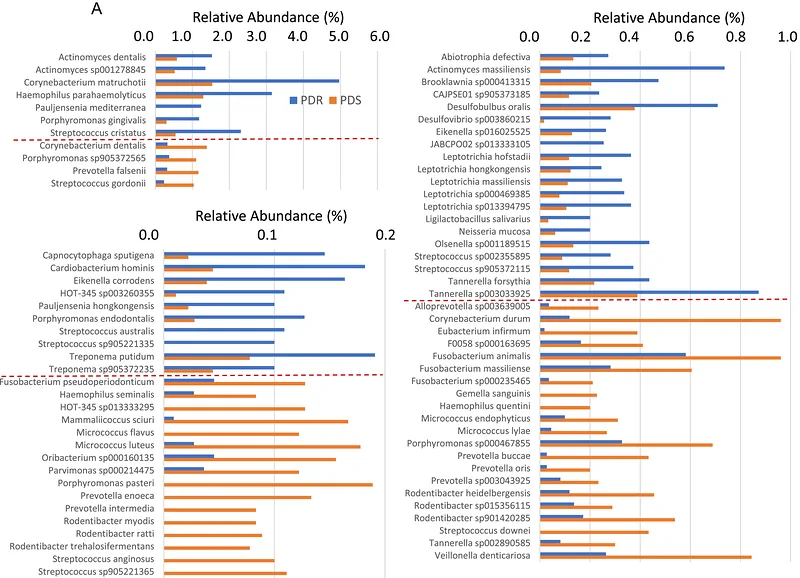

**Figure:**
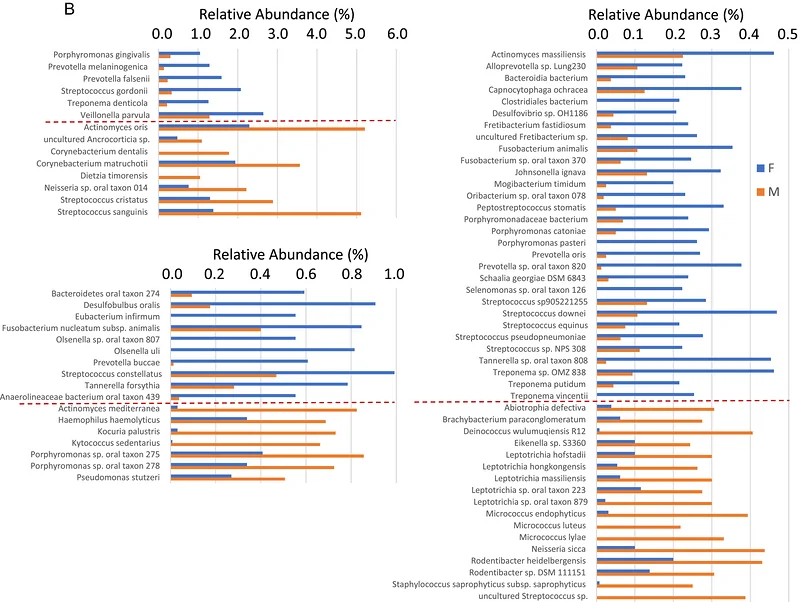

**Figure:**
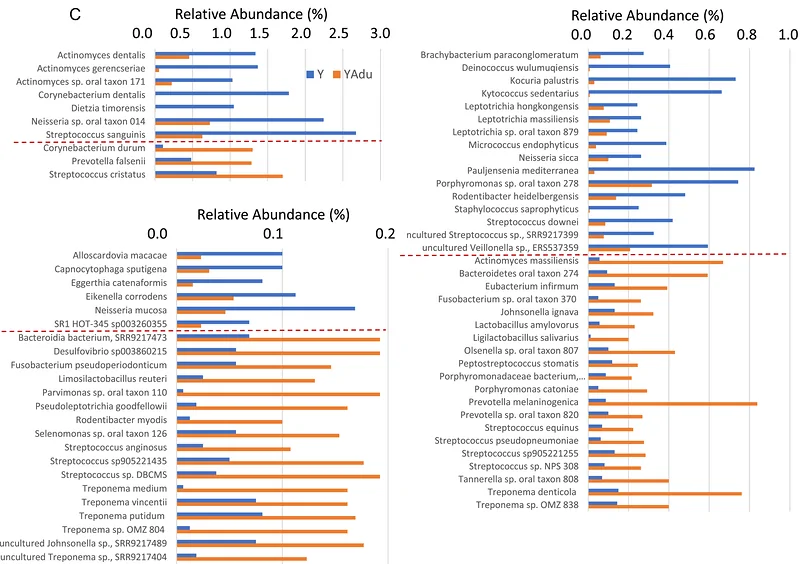

Sex‐associated major differences in microbiome components are shown in Figure 6B. This comparison revealed that female nonhuman primates displayed a mixed array of commensals and pathobionts, including *Porphyromonas, Fusobacteria, Tannerella*, and *Treponema*. While the male samples contained elevated levels of some species considered pathogens in human disease, the frequency of elevations in *Leptotrichia* and *Rodentibacter* species was a striking feature.

Figure 6C provides a summary of a similar comparison based on age differences in this younger group of nonhuman primates. First, the largest number of differences was identified with elevated levels in the YAdu samples. Second, the majority of the elevated levels in the Y group reflected species generally considered as commensal oral bacteria. Finally, those species at greater abundance in the YAdu nonhuman primates represented a variety of purported pathogens in humans, including *Porphyromonas, Fusobacteria, Tannerella*, and *Treponema* species. Thus, the distribution of these differences appeared to support the notion that in these young, orally healthy nonhuman primates, pathogenic species routinely exist. They were increased in PDS and YAdu nonhuman primates, although their relative levels were rather similar in males and females.

### 3.4 Microbiome Relationship to Clinical Measures in Young Nonhuman Primates

While these represent young, healthy nonhuman primates, some differences in both the extent of BOP and PPD were noted across this population. As such, we explored relationships between individual microbiome members and these clinical measures. Figure S4A–C summarizes the OTUs that demonstrated a significant correlation between relative abundance and one or both of the clinical measures. Included in this analysis were only those OTUs in which levels were detectable in at least 50% of the animal samples. Figure S4A identified the taxa showing a significant positive correlation with the mouth mean BOP index. This included multiple *Actinomyces* species and *Prev. falsenii*. Figure S4B provides a similar display of OTU levels with significant correlations to the percent of sites with any BOP. While *Prev. falsenii* was again in this subset; an interesting observation was that the other microbial correlations of *Arachnia* (Gram‐positive anaerobe), *P. gingivicola* (Gram‐positive coccus), and *Treponema* sp. OMZ 838 (isolated from human ANUG) are human oral colonizers, in some cases related to periodontal diseases (Al Kawas et al. 2021; Beall et al. 2018; Chen et al. 2024). Figure S3C depicts the relationship between various *Fusobacterium* species, considered an important oral pathobiont, and a measure of periodontal pocketing. Nevertheless, the striking feature of this analysis of microbiome members and clinical features in these younger nonhuman primates was the very limited number of OTUs showing a significant correlation to the clinical measures, suggesting that in these younger nonhuman primates, the host–bacterial interactions have not transitioned to a greater risk for development of the features of immunoinflammatory lesions.

## 4 Discussion

Nonhuman primates of numerous genera and species have been shown to develop gingivitis and periodontitis with clinical and demographic features similar to humans (Ebersole et al. 2025; Eke et al. 1998; Hajishengallis 2023; Krygier et al. 1973; Schou et al. 1993). An array of studies has shown nonhuman primate similarities to humans in the features of the oral microbiome, both in health and in periodontal lesions (Colombo et al. 2017; Kirakodu et al. 2019; Takehara et al. 2019). Also, due to the close genetic relationship of nonhuman primates and humans, many aspects of the innate immune, inflammatory, and adaptive immune system have been identified to be conserved within healthy and diseased periodontal tissues between humans and nonhuman primates (Ebersole et al. 2017; Ebersole et al. 2025). Beyond these observations of naturally occurring disease, nonhuman primates have been utilized as a preclinical model of experimental periodontitis, elicited by ligature placement around teeth to initiate localized periodontal lesions. Thus, this preclinical disease model is well‐positioned to elucidate novel biologic‐based preventive and therapeutic approaches to controlling periodontitis as a chronic infection and immunoinflammatory disease.

We have reported previously on investigations using the colony of *Macaca mulatta* housed through a National Primate Research Center on the island of Cayo Santiago and at the Caribbean Primate Research Center (Ebersole et al. 2025). Beyond studies to detail the oral microbiome and gingival mucosal responses to the microbial insult, this animal cohort provided the ability to evaluate variations in these oral features related to sex (Ebersole et al. 2023c) and heritability (e.g., matriline) features (Ebersole et al. 2023a). In this analysis, the oral microbiomes of younger (10–19 human years) nonhuman primates were described to differentiate matriline (an indicator of heritability), sex, and age. Through historical skeletal analysis and living descendants, we have previously described matrilines that demonstrate a pattern of periodontal disease susceptibility or resistance. This concept is consistent with human studies supporting some genetic contribution to periodontitis (Shaddox et al. 2021). Additionally, epidemiological results from human populations and our results from nonhuman primates support an elevated prevalence of periodontitis in male nonhuman primates, particularly with aging.

A holistic assessment of the microbiome similarities/differences suggested two primary conclusions in this younger group of orally healthy nonhuman primates. First, at the microbiome level of phyla, orders, and families, the ecology was relatively similar across sexes, matrilines, and age groups. However, at the genus level, microbiome differences in matrilines, sex, or age were observed. Of interest was that the principal differential genus proportions were similar between matriline and age comparisons, but somewhat unique with sex comparisons. Nevertheless, these genus differences encompassed microorganisms generally considered commensals in human studies, albeit colonization by *Fusobacterium, Tannerella*, and *Treponema* genera showed some variation. These sex‐based differences, even in young individuals, are consistent with recent data from Kumar and colleagues (Nikam et al. 2026). whereby they delineated substantive differences in the oral microbiomes of younger humans, albeit aging dramatically modified these differences

The second, broader observation was the rather extensive species variation across these nonhuman primates. Nevertheless, we could define a “core microbial species” pattern whereby one‐third or more of the samples showed detectable subgingival levels of the particular species. This core included species across the *Actinobiota* (*n* = 19)*, Bacteroidota* (*n* = 10)*, Desulfobacterota, Firmicutes/Bacillota* (*n* = 17)*, Fusobacteriota* (*n* = 16)*, Proteobacteriota* (*n* = 10), and *Spirochaetota* phyla. However, drilling down into the ecology of lower‐abundance microorganisms, substantial variation of individual species across all phyla was observed. Additionally, the levels between sexes showed a greater number of significant differences compared to the data contrasting the matriline samples, and even compared with age‐based differences. This was somewhat unexpected, as these nonhuman primates organize in matriline‐based social structures. They reside in larger multi‐generational family units in independent corrals; thus, the hypothesis was that the PDR or PDS matriline derivation would demonstrate greater differences. This conjecture was also related to an expectation that even in this younger age group of nonhuman primates, characteristics of the oral microbiome might presage the greater frequency and severity of disease observed throughout the PDS matrilines. The OTUs elevated in the PDR samples appeared skewed toward many Gram‐positive bacteria and Gram‐negative commensals, such as *Leptotrichia* species; however, purported human periodontal pathogens (e.g., *Treponema, Tannerella, Desulfobulbus, Desulfovibrio* species) could contribute to pathogenic changes later in life. In contrast, in the PDS nonhuman primates, elevations in numerous *Porphyromonas, Prevotella*, and *Fusobacterium* species were observed. The array of *Rodentibacter* species that were elevated has been identified as major infection agents in rodents (Hansen et al. 2019), and gut or uropathogens in humans (Prabhu and Harshan 2026). Comparison of microbiomes by sex showed that female nonhuman primates displayed a mixed array of commensals and pathobionts, including *Porphyromonas, Fusobacteria, Tannerella*, and *Treponema*. While the male samples contained many of these same pathogenic species, specific elevations in *Leptotrichia* and *Rodentibacter* species were discerned. Additionally, even in this relatively young group of nonhuman primates, age differences were noted with a larger number of elevations in abundance in the YAdu samples, while the Y group elevations primarily represented commensal bacteria. The species elevated in the YAdu group were consistent with human pathogens, including species of *Porphyromonas, Fusobacteria, Tannerella*, and *Treponema*. Thus, the observations of increased prevalence and severity of periodontitis in adult male humans and nonhuman primates may reflect differences in the early‐life establishment of the oral microbiome ecology. Moreover, multiple reports have described sex‐based diversity in oral biofilm microbial composition (Del Pinto et al. 2024), sex hormone effects on the oral microbiome (Cornejo Ulloa et al. 2021), and likely interactions between the oral microbiome and host characteristics related to sex (Liu et al. 2023). Additionally, diverse types of immune system‐based sex variations have been reported with vaccine responses (Fischinger et al. 2019; Taneja 2018), autoimmune diseases (Kim et al. 2025; Moulton 2018), and susceptibility to mucosal infections (Alrashdan et al. 2023; Deltourbe et al. 2022; Tang et al. 2025) that could impact the response to the oral microbiome constituents. We have also identified within the gingival transcriptome distinctive sex differences in the portfolio of innate immune, inflammatory, and adaptive immune response features in an experimental periodontitis model (Ebersole et al. 2021; Ebersole et al. 2023b; Ebersole et al. 2023c; Ebersole et al. 2008a,b). The potential translational importance of these findings is related to epidemiological reports describing the increased extent and severity of periodontitis in males that do not appear to have simple behavioral explanations affiliated with oral hygiene practices and/or solicitation of needed dental services. Conversely, suspected fundamental biological differences that are sex‐based in the composition of the autochthonous oral ecology and the host response to this ecology remain ill‐defined. The variations in early‐life gingival tissue programming and responses to microbiome dissimilarities could contribute to the magnitude of differences in sex‐based disease susceptibility that occur later in life.

## 5 Conclusion

This investigation elucidated characteristics of the oral microbiome using healthy younger nonhuman primates as a preclinical model displaying clinical, microbiological, and immunological features similar to the human oral cavity. Limitations of the study include a relatively small number of nonhuman primates when stratified into matriline, sex, or age subgroups. However, while the availability and cost of this large animal model are challenging for study design, the substantive similarities with the human complex oral cavity environment enable this preclinical model to aid in providing a straightforward translational component in understanding human disease. Additionally, only one pooled subgingival plaque sample was characterized from each animal. The clinical findings supported the overall oral health of each animal; however, there were clear differences in the frequency and distribution of sites with BOP, with less variation in PPD. Thus, these samples may not accurately describe the within‐subject variation in the microbiome from various subgingival sites in the oral cavity. Nevertheless, this implementation of microbiome characterization is quite similar to studies utilizing human samples collected from single or pooled subgingival sites. Finally, as this is a cross‐sectional study, samples of the younger nonhuman primates (e.g., ∼10–12 human years) to the older nonhuman primates (∼13–19 human years) are independent; they may not accurately depict the longitudinal alterations that could occur in the nonhuman primates and in humans over these early life decades.

## Conflicts of Interest

The authors state no conflict of interest with any of the aspects of this study and report.

## Supporting information




**Table S1**: Top 100 relative abundance species of bacteria in oral microbiomes of *Macaca mulatta* ranked based upon mean levels across all comparisons.
**Figure S1**: Alpha and Beta diversity data of the microbial species for individual animals and stratified by matriline, sex, or age of the nonhuman primates.
**Figure S2**: Relative abundance of microbial orders in the oral microbiome stratified by matriline (PDR/PDS), sex (female/male), and age (young, young adult). The asterisk denotes statistically significantly different at *p* < 0.05.
**Figure S3**: Relative abundance of microbial families in the oral microbiome stratified by matriline (PDR/PDS), sex (female/male), and age (young, young adult). The values denote the mean relative abundance across all 30 samples.
**Figure S4**: Correlation displays of relative abundance of a particular species with mouth mean BOP (A), percentage of sites with BOP (B), and either mouth mean PPD or percentage of sites with PPD >2 mm (C).

## Data Availability

The data that support the findings of this study are available from the corresponding author upon reasonable request.
