# Supplementary material for: Characteristics of the Health‐Associated Oral Microbiome in Young Nonhuman Primates
Source: Mol Oral Microbiol. 2026 May 16;41(5):266–79. doi: 10.1111/omi.70033 (PMC13535889; doi:10.1111/omi.70033)
Supplement: Supplementary file 1 [file OMI-41-266-s001.docx]

**Supplemental Table 1:** Top 100 relative abundance species of bacteria in oral microbiomes of *Macaca mulatta* ranked based upon mean levels across all comparisons.

| **Phylum** | **Order** | **Family** | **Genus** | **Species** | **Matriline** | | **Sex** | | **Age** | |
| --- | --- | --- | --- | --- | --- | --- | --- | --- | --- | --- |
|  | | | | | **PDR** | **PDS** | **F** | **M** | **Y** | **YAdu** |
| Actinobacteriota | Actinomycetales | Actinomycetaceae | Actinomyces | Actinomyces johnsonii strain=CCUG 34287 | 8.56 | 10.66 | 8.75 | 10.76 | 8.84 | 11.12 |
| Actinobacteriota | Actinomycetales | Actinomycetaceae | Actinomyces | Actinomyces sp ZJ308 strain=ZJ308 | 3.87 | 5.75 | 4.25 | 5.68 | 3.83 | 6.52 |
| Actinobacteriota | Mycobacteriales | Mycobacteriaceae | Corynebacterium | Corynebacterium matruchotii strain=ATCC 14266 | 4.96 | 1.54 | 1.94 | 3.57 | 2.76 | 2.94 |
| Actinobacteriota | Propionibacteriales | Propionibacteriaceae | Arachnia | Propionibacterium sp | 3.16 | 1.87 | 2.43 | 2.31 | 1.94 | 2.88 |
| Actinobacteriota | Actinomycetales | Actinomycetaceae | Actinomyces | Actinomyces viscosus strain=NCTC10951 | 1.69 | 2.92 | 2.31 | 2.57 | 1.99 | 3.02 |
| Actinobacteriota | Propionibacteriales | Propionibacteriaceae | Arachnia | Pseudopropionibacterium propionicum  strain=NCTC12967 | 2.84 | 1.78 | 2.18 | 2.18 | 2.29 | 2.05 |
| Actinobacteriota | Propionibacteriales | Propionibacteriaceae | Arachnia | Propionibacteriaceae bacterium | 2.87 | 1.68 | 2.35 | 1.96 | 2.03 | 2.27 |
| Actinobacteriota | Actinomycetales | Actinomycetaceae | Actinomyces | Actinomyces naeslundii strain=NCTC 10301 | 1.92 | 2.06 | 1.49 | 2.42 | 2.09 | 1.89 |
| Actinobacteriota | Actinomycetales | Actinomycetaceae | Actinomyces | Actinomyces sp900323545 | 1.70 | 1.83 | 1.43 | 2.07 | 1.44 | 2.21 |
| Actinobacteriota | Actinomycetales | Actinomycetaceae | Actinomyces | Actinomyces sp  oral taxon 170 str F0386 | 1.18 | 2.04 | 1.63 | 1.79 | 1.31 | 2.22 |
| Proteobacteria | Burkholderiales | Neisseriaceae | Neisseria | Neisseria sp  oral taxon 014 str F0314 | 1.25 | 1.76 | 0.76 | 2.22 | 2.24 | 0.73 |
| Actinobacteriota | Actinomycetales | Actinomycetaceae | Peptidiphaga | Peptidiphaga gingivicola strain=BA112 | 1.81 | 1.18 | 1.00 | 1.76 | 1.25 | 1.63 |
| Proteobacteria | Enterobacterales | Pasteurellaceae | Aggregatibacter | A. actinomycetemcomitans strain=SL7469 | 1.26 | 1.43 | 1.78 | 1.03 | 1.22 | 1.55 |
| Actinobacteriota | Actinomycetales | Actinomycetaceae | Actinomyces | Actinomyces oris strain=WE8B-23 | 1.77 | 0.98 | 0.52 | 1.91 | 1.61 | 0.88 |
| Proteobacteria | Enterobacterales | Pasteurellaceae | Aggregatibacter | Aggregatibacter actinomycetemcomitans strain=HK | 0.90 | 1.18 | 1.50 | 0.73 | 0.96 | 1.22 |
| Proteobacteria | Enterobacterales | Pasteurellaceae | Haemophilus | Haemophilus parahaemolyticus strain=CCUG 3716 | 1.55 | 0.72 | 0.78 | 1.24 | 1.13 | 0.92 |
| Proteobacteria | Enterobacterales | Pasteurellaceae | Haemophilus | H. paraphrohaemolyticus strain=CCUG 3718 | 1.60 | 0.57 | 0.82 | 1.08 | 0.96 | 0.96 |
| Actinobacteriota | Propionibacteriales | Propionibacteriaceae | Arachnia | Pseudopropionibacterium massiliense  strain=Marseille-P6184 | 1.22 | 0.86 | 0.75 | 1.19 | 1.26 | 0.68 |
| Actinobacteriota | Actinomycetales | Actinomycetaceae | Actinomyces | Actinomyces dentalis DSM 19115 strain=DSM 19115 | 1.53 | 0.58 | 1.26 | 0.68 | 1.34 | 0.45 |
| Firmicutes | Staphylococcales | Gemellaceae | Gemella | Gemella morbillorum strain=NCTC11323 | 1.04 | 0.92 | 0.94 | 0.99 | 1.00 | 0.92 |
| Actinobacteriota | Actinomycetales | Actinomycetaceae | Actinomyces | Actinomyces oris strain=S64C | 0.55 | 1.19 | 0.77 | 1.09 | 1.04 | 0.84 |
| Actinobacteriota | Actinomycetales | Actinomycetaceae | Actinomyces | Actinomyces oris strain=CCUG 34288 | 0.94 | 0.86 | 0.79 | 0.97 | 1.05 | 0.69 |
| Actinobacteriota | Mycobacteriales | Mycobacteriaceae | Corynebacterium | Corynebacterium dentalis strain=Marseille-P4122 | 0.33 | 1.39 | 0.01 | 1.78 | 1.78 | 0.01 |
| Actinobacteriota | Actinomycetales | Actinomycetaceae | Actinomyces | Actinomyces sp oral taxon 414 strain=F0588 | 1.35 | 0.52 | 1.15 | 0.58 | 1.17 | 0.43 |
| Bacteroidota | Bacteroidales | Bacteroidaceae | Prevotella | Prevotella falsenii strain=JCM 15124 | 0.32 | 1.16 | 1.59 | 0.23 | 0.48 | 1.28 |
| Fusobacteriota | Fusobacteriales | Fusobacteriaceae | Fusobacterium | Fusobacterium canifelinum strain=FDAARGOS | 0.66 | 0.95 | 1.05 | 0.68 | 0.74 | 0.96 |
| Actinobacteriota | Actinomycetales | Actinomycetaceae | Peptidiphaga | uncultured Ancrocorticia sp | 1.09 | 0.65 | 0.48 | 1.09 | 0.81 | 0.83 |
| Bacteroidota | Bacteroidales | Porphyromonadaceae | Porphyromonas | uncultured Porphyromonas sp | 0.37 | 1.11 | 0.89 | 0.78 | 0.88 | 0.77 |
| Firmicutes | Lactobacillales | Streptococcaceae | Streptococcus | Streptococcus oralis strain=DD25 | 0.82 | 0.73 | 0.95 | 0.62 | 0.69 | 0.85% |
| Proteobacteria | Enterobacterales | Pasteurellaceae | Aggregatibacter | Aggregatibacter kilianii strain=PN | 0.87 | 0.70 | 0.77 | 0.76 | 0.86 | 0.65 |
| Actinobacteriota | Actinomycetales | Actinomycetaceae | Actinomyces | Actinomyces gerencseriae strain=DSM 6844 | 0.08 | 1.21 | 1.67 | 0.06 | 1.37 | 0.05 |
| Fusobacteriota | Fusobacteriales | Fusobacteriaceae | Fusobacterium | F. nucleatum subsp  polymorphum strain=KCOM 1232 | 0.55 | 0.84 | 0.88 | 0.61 | 0.70 | 0.77 |
| Fusobacteriota | Fusobacteriales | Fusobacteriaceae | Fusobacterium | Fusobacterium hwasookii strain=KCOM 1249 | 0.51 | 0.81 | 0.94 | 0.50 | 0.55 | 0.88 |
| Fusobacteriota | Fusobacteriales | Fusobacteriaceae | Fusobacterium | F. nucleatum subsp  polymorphum strain=NCTC10562 | 0.58 | 0.76 | 0.86 | 0.55 | 0.56 | 0.85 |
| Bacteroidota | Bacteroidales | Porphyromonadaceae | Porphyromonas | Porphyromonas gingivalis  strain=ATCC 33277 | 1.18 | 0.30 | 1.05 | 0.30 | 0.53 | 0.77 |
| Proteobacteria | Enterobacterales | Pasteurellaceae | Aggregatibacter | A. actinomycetemcomitans strain=DSM 8324 | 0.64 | 0.69 | 0.88 | 0.49 | 0.61 | 0.75 |
| Actinobacteriota | Actinomycetales | Actinomycetaceae | Actinomyces | Actinomyces sp  oral taxon 171 str F0337 | 0.80 | 0.59 | 0.55 | 0.77 | 1.03 | 0.22 |
| Firmicutes | Veillonellales | Veillonellaceae | Veillonella | Veillonella parvula strain=DSM 2008 | 0.41 | 0.83 | 0.95 | 0.44 | 0.84 | 0.45 |
| Firmicutes | Lactobacillales | Streptococcaceae | Streptococcus | Streptococcus mitis ATCC strain=ATCC 6249 | 0.61 | 0.66 | 0.78 | 0.53 | 0.46 | 0.86 |
| Actinobacteriota | Actinomycetales | Micrococcaceae | Rothia | Rothia dentocariosa strain=ATCC 17931 | 0.57 | 0.71 | 0.46 | 0.82 | 0.73 | 0.57 |
| Bacteroidota | Bacteroidales | Porphyromonadaceae | Porphyromonas | Porphyromonas sp  oral taxon 275 strain=W7780 | 0.62 | 0.68 | 0.41 | 0.86 | 0.83 | 0.45 |
| Actinobacteriota | Actinomycetales | Actinomycetaceae | Peptidiphaga | Actinobaculum sp oral taxon 183 str  F0552 | 0.84 | 0.48 | 0.65 | 0.58 | 0.56 | 0.68 |
| Actinobacteriota | Mycobacteriales | Mycobacteriaceae | Corynebacterium | Corynebacterium durum strain=DSM 45333 | 0.12 | 0.96 | 0.75 | 0.56 | 0.11 | 1.30 |
| Fusobacteriota | Fusobacteriales | Fusobacteriaceae | Fusobacterium | Fusobacterium nucleatum strain=W1481 | 0.53 | 0.68 | 0.75 | 0.52 | 0.64 | 0.60 |
| Fusobacteriota | Fusobacteriales | Fusobacteriaceae | Fusobacterium | Fusobacterium nucleatum subsp  animalis | 0.54 | 0.64 | 0.85 | 0.40 | 0.49 | 0.73 |
| Firmicutes | Veillonellales | Veillonellaceae | Veillonella | Veillonella denticariosi JCM 15641 strain=JCM 15641 | 0.26 | 0.84 | 0.83 | 0.46 | 0.68 | 0.56 |
| Firmicutes | Lactobacillales | Streptococcaceae | Streptococcus | Streptococcus oralis strain=DD24 | 0.62 | 0.58 | 0.62 | 0.58 | 0.48 | 0.75% |
| Bacteroidota | Bacteroidales | Tannerellaceae | Tannerella | Tannerella sp  oral taxon HOT-286 strain=W11666 | 0.87 | 0.39 | 0.58 | 0.57 | 0.51 | 0.65% |
| Fusobacteriota | Fusobacteriales | Fusobacteriaceae | Fusobacterium | F. nucleatum subsp  nucleatum strain=ATCC 25586 | 0.43 | 0.64 | 0.71 | 0.44 | 0.51 | 0.62 |
| Firmicutes | Lactobacillales | Streptococcaceae | Streptococcus | Streptococcus constellatus | 0.52 | 0.56 | 0.82 | 0.32 | 0.47 | 0.64 |
| Firmicutes | Veillonellales | Veillonellaceae | Veillonella | Veillonella parvula strain=SKV38 | 0.41 | 0.65 | 0.75 | 0.40 | 0.72 | 0.36 |
| Actinobacteriota | Mycobacteriales | Mycobacteriaceae | Dietzia | Dietzia timorensis strain=ID05-A0528 | 0.55 | 0.60 | 0.01 | 1.05 | 1.05 | 0.01 |
| Firmicutes | Veillonellales | Veillonellaceae | Veillonella | Veillonella rodentium strain=NCTC12018 | 0.18 | 0.80 | 0.77 | 0.40 | 0.63 | 0.49 |
| Firmicutes | Lactobacillales | Streptococcaceae | Streptococcus | Streptococcus sanguinis  NCTC 7863 strain=SK1 | 0.33 | 0.72 | 0.55 | 0.59 | 0.81 | 0.27 |
| Desulfobacterota | Desulfobulbales | Desulfobulbaceae | Desulfobulbus | Desulfobulbus oralis strain=HOT041/ORNL | 0.71 | 0.38 | 0.91 | 0.18 | 0.42 | 0.61 |
| Bacteroidota | Bacteroidales | Porphyromonadaceae | Porphyromonas | Porphyromonas sp oral taxon 278 str W7784 | 0.33 | 0.69 | 0.34 | 0.73 | 0.74 | 0.32 |
| Actinobacteriota | Actinomycetales | Actinomycetaceae | Pauljensenia | Actinomyces mediterranea strain=Marseille-P3257 | 1.24 | 0.00 | 0.03 | 0.83 | 0.83 | 0.03 |
| Fusobacteriota | Fusobacteriales | Fusobacteriaceae | Fusobacterium | Fusobacterium massiliense strain=Marseille-P2749 | 0.28 | 0.61 | 0.45 | 0.51 | 0.51 | 0.45 |
| Actinobacteriota | Actinomycetales | Actinomycetaceae | Actinomyces | Actinomyces oris strain=T14V | 0.64 | 0.34 | 0.22 | 0.65 | 0.61 | 0.26 |
| Fusobacteriota | Fusobacteriales | Leptotrichiaceae | Leptotrichia | Leptotrichia trevisanii DSM 22070 strain=DSM 22070 | 0.63 | 0.32 | 0.40 | 0.47 | 0.44 | 0.43 |
| Fusobacteriota | Fusobacteriales | Fusobacteriaceae | Fusobacterium | F. nucleatum subsp  vincentii strain=ATCC 49256 | 0.38 | 0.41 | 0.55 | 0.28 | 0.29 | 0.54 |
| Firmicutes | Veillonellales | Veillonellaceae | Veillonella | uncultured Veillonella sp | 0.26 | 0.52 | 0.54 | 0.33 | 0.59 | 0.21 |
| Firmicutes | Staphylococcales | Gemellaceae | Gemella | Gemella haemolysans strain=ATCC 10379 | 0.46 | 0.37 | 0.37 | 0.43 | 0.46 | 0.33 |
| Actinobacteriota | Actinomycetales | Micrococcaceae | Kocuria | Kocuria palustris strain=TAGA27 | 0.53 | 0.35 | 0.03 | 0.73 | 0.73 | 0.03 |
| Proteobacteria | Burkholderiales | Neisseriaceae | Eikenella | Eikenella longinqua strain=NML02-A-017 | 0.33 | 0.42 | 0.37 | 0.40 | 0.33 | 0.46 |
| Firmicutes | Lactobacillales | Streptococcaceae | Streptococcus | Streptococcus gordonii strain=CCUG 33482 | 0.13 | 0.54 | 0.72 | 0.11 | 0.41 | 0.35 |
| Proteobacteria | Enterobacterales | Pasteurellaceae | Rodentibacter | Pseudomonas stutzeri strain=NCTC11607 | 0.17 | 0.54 | 0.27 | 0.51 | 0.62 | 0.13 |
| Actinobacteriota | Actinomycetales | Actinomycetaceae | Actinomyces | Actinomyces massiliensis strain=4401292 | 0.74 | 0.08 | 0.46 | 0.23 | 0.06 | 0.67 |
| Actinobacteriota | Coriobacteriales | Atopobiaceae | Olsenella | Olsenella uli DSM strain=DSM 7084 | 0.28 | 0.42 | 0.82 | 0.00 | 0.47 | 0.24 |
| Bacteroidota | Flavobacteriales | Flavobacteriaceae | Capnocytophaga | Capnocytophaga endodontalis strain=ChDC OS43 | 0.45 | 0.31 | 0.26 | 0.45 | 0.34 | 0.40 |
| Actinobacteriota | Actinomycetales | Dermatophilaceae | Kytococcus | Kytococcus sedentarius  strain=DSM 20547 | 0.39 | 0.36 | 0.01 | 0.66 | 0.66 | 0.01 |
| Firmicutes | Lactobacillales | Streptococcaceae | Streptococcus | Streptococcus gordonii strain=DD07 | 0.11 | 0.49 | 0.65 | 0.11 | 0.40 | 0.28 |
| Bacteroidota | Bacteroidales | Paludibacteraceae | F0058 | Bacteroidetes oral taxon 274 str  F0058 | 0.16 | 0.41 | 0.59 | 0.09 | 0.09 | 0.59 |
| Firmicutes | Lactobacillales | Streptococcaceae | Streptococcus | Streptococcus oralis strain=FDAARGOS | 0.39 | 0.26 | 0.40 | 0.23 | 0.18 | 0.47 |
| Firmicutes | Lactobacillales | Streptococcaceae | Streptococcus | Streptococcus cristatus strain=A53 | 0.59 | 0.12 | 0.19 | 0.39 | 0.23 | 0.38 |
| Actinobacteriota | Propionibacteriales | Propionibacteriaceae | Brooklawnia | Propionibacterium sp  oral taxon 192 str F0372 | 0.47 | 0.21 | 0.25 | 0.36 | 0.35 | 0.25 |
| Actinobacteriota | Actinomycetales | Actinomycetaceae | Actinomyces | Actinomyces oris strain=P6N | 0.31 | 0.33 | 0.00 | 0.59 | 0.38 | 0.26 |
| Fusobacteriota | Fusobacteriales | Leptotrichiaceae | Leptotrichia | Leptotrichia buccalis C-1013-b strain=DSM 1135 | 0.39 | 0.25 | 0.25 | 0.35 | 0.33 | 0.27 |
| Proteobacteria | Enterobacterales | Pasteurellaceae | Rodentibacter | Rodentibacter heidelbergensis strain=Ac69 | 0.12 | 0.46 | 0.20 | 0.43 | 0.48 | 0.14 |
| Fusobacteriota | Fusobacteriales | Leptotrichiaceae | Leptotrichia | Leptotrichia sp oral taxon 212 strain=W10393 | 0.27 | 0.33 | 0.22 | 0.38 | 0.34 | 0.27 |
| Chloroflexota | Anaerolineales | Anaerolineaceae | Flexilinea | Anaerolineaceae oral taxon 439 strain=W11661 | 0.33 | 0.23 | 0.55 | 0.04 | 0.26 | 0.28 |
| Actinobacteriota | Coriobacteriales | Atopobiaceae | Olsenella | Olsenella sp oral taxon 807 strain=F0089 | 0.44 | 0.13 | 0.55 | 0.00 | 0.10 | 0.43 |
| Bacteroidota | Bacteroidales | Bacteroidaceae | Prevotella | Prevotella buccae strain=ATCC 33574 | 0.03 | 0.43 | 0.61 | 0.01 | 0.23 | 0.35 |
| Spirochaetota | Treponematales | Treponemataceae | Treponema | Treponema denticola strain=ATCC 35405 | 0.21 | 0.29 | 0.47 | 0.09 | 0.08 | 0.48 |
| Spirochaetota | Treponematales | Treponemataceae | Treponema | Treponema sp strain=OMZ 838 (ATCC 700772) | 0.22 | 0.28 | 0.46 | 0.09 | 0.14 | 0.40 |
| Firmicutes | Lactobacillales | Streptococcaceae | Streptococcus | Streptococcus cristatus strain=BCC51 | 0.44 | 0.14 | 0.21 | 0.29 | 0.14 | 0.39 |
| Firmicutes | Lactobacillales | Streptococcaceae | Streptococcus | Streptococcus downei strain=NCTC 11391 | 0.00 | 0.43 | 0.47 | 0.11 | 0.42 | 0.08 |
| Proteobacteria | Burkholderiales | Burkholderiaceae | Lautropia | Lautropia mirabilis strain=NCTC12852 | 0.34 | 0.19 | 0.25 | 0.24 | 0.32 | 0.15 |
| Firmicutes | Peptostreptococcales | Anaerovoracaceae | Eubacterium | Eubacterium infirmum strain=NCTC12940 | 0.02 | 0.39 | 0.55 | 0.00 | 0.13 | 0.39 |
| Firmicutes | Lactobacillales | Streptococcaceae | Streptococcus | Streptococcus cristatus strain=ATCC 51100 | 0.47 | 0.08 | 0.12 | 0.33 | 0.20 | 0.27 |
| Bacteroidota | Flavobacteriales | Flavobacteriaceae | Capnocytophaga | Capnocytophaga ochraceastrain=DSM 7271 | 0.22 | 0.25 | 0.38 | 0.13 | 0.19 | 0.30 |
| Firmicutes | Lactobacillales | Streptococcaceae | Streptococcus | Streptococcus cristatus strain=DD08 | 0.40 | 0.11 | 0.15 | 0.28 | 0.11 | 0.36 |
| Firmicutes | Lactobacillales | Streptococcaceae | Streptococcus | Streptococcus sanguinis strain=SK355 | 0.26 | 0.22 | 0.00 | 0.43 | 0.43 | 0.00 |
| Firmicutes | Lachnospirales | Lachnospiraceae | Johnsonella | Johnsonella ignava  strain=ATCC 51276 | 0.21 | 0.22 | 0.32 | 0.13 | 0.13 | 0.32 |
| Bacteroidota | Bacteroidales | Tannerellaceae | Tannerella | Tannerella sp  oral taxon 808 | 0.08 | 0.30 | 0.45 | 0.03 | 0.07 | 0.40 |
| Firmicutes | Lactobacillales | Streptococcaceae | Streptococcus | Streptococcus sanguinis strain=SK49 | 0.31 | 0.18 | 0.00 | 0.41 | 0.39 | 0.03 |
| Bacteroidota | Bacteroidales | Tannerellaceae | Tannerella | Tannerella forsythia strain=ATCC 43037 | 0.27 | 0.17 | 0.33 | 0.11 | 0.19 | 0.23 |
| Fusobacteriota | Fusobacteriales | Leptotrichiaceae | Leptotrichia | Leptotrichia hofstadii strain=JCM16775 | 0.36 | 0.12 | 0.10 | 0.30 | 0.26 | 0.15 |
| Proteobacteria | Enterobacterales | Pasteurellaceae | Rodentibacter | Rodentibacter sp strain=DSM 11115 | 0.14 | 0.29 | 0.14 | 0.31 | 0.42 | 0.00 |
| Deinococcota | Deinococcales | Deinococcaceae | Deinococcus | Deinococcus wulumuqiensis strain=R12 | 0.22 | 0.23 | 0.01 | 0.41 | 0.41 | 0.01 |
| Firmicutes | Lactobacillales | Streptococcaceae | Streptococcus | uncultured Streptococcus sp | 0.37 | 0.12 | 0.00 | 0.39 | 0.33 | 0.08 |
| Fusobacteriota | Fusobacteriales | Fusobacteriaceae | Fusobacterium | Fusobacterium animalis | 0.05 | 0.32 | 0.35 | 0.11 | 0.15 | 0.30 |
| Firmicutes | Lactobacillales | Streptococcaceae | Streptococcus | Streptococcus oralis strain=JPIIBV3 | 0.17 | 0.24 | 0.15 | 0.28 | 0.25 | 0.18% |
| Actinobacteriota | Actinomycetales | Micrococcaceae | Micrococcus | Micrococcus endophyticus strain=DSM 17945 | 0.10 | 0.31 | 0.03 | 0.39 | 0.39 | 0.04 |
| Fusobacteriota | Fusobacteriales | Leptotrichiaceae | Leptotrichia | Leptotrichia sp013394795 | 0.36 | 0.11 | 0.12 | 0.28 | 0.24 | 0.16 |
| Firmicutes | Lactobacillales | Streptococcaceae | Streptococcus | Streptococcus sanguinis strain=2908 | 0.22 | 0.21 | 0.08 | 0.32 | 0.26 | 0.16 |
| Bacteroidota | Bacteroidales | Bacteroidaceae | Prevotella | Prevotella melaninogenica strain=F0091 | 0.15 | 0.22 | 0.41 | 0.03 | 0.05 | 0.38 |
| Firmicutes | Lactobacillales | Streptococcaceae | Streptococcus | Streptococcus sp905221255 | 0.19 | 0.21 | 0.28 | 0.13 | 0.13 | 0.28 |
| Fusobacteriota | Fusobacteriales | Leptotrichiaceae | Leptotrichia | Leptotrichia massiliensis strain=Marseille-P3007 | 0.33 | 0.11 | 0.06 | 0.30 | 0.26 | 0.11 |

**Supplemental Figure 1:** Alpha and Beta diversity data of the microbial species for individual animals and stratified by matriline, sex, or age of the nonhuman primates.

**Supplemental Figure 2:** Relative abundance of microbial orders in the oral microbiome stratified by matriline (PDR/PDS), sex (female/male), and age (young, young adult). The asterisk denotes statistically significantly different at p<0.05.

**Supplemental Figure 3:** Relative abundance of microbial families in the oral microbiome stratified by matriline (PDR/PDS), sex (female/male), and age (young, young adult). The values denote the mean relative abundance across all 30 samples.

**Supplemental Figure 4:** Correlation displays of relative abundance of a particular species with mouth mean BOP (**A**), percentage of sites with BOP (**B**), and either mouth mean PPD or percentage of sites with PPD >2 mm (**C**).
